# National Competence Based Catalogue of Learning Objectives for Undergraduate Medical Education (NKLM) – process description over the last 15 years

**DOI:** 10.3205/zma001862

**Published:** 2026-06-15

**Authors:** Jacqueline Jennebach, Julian Özkaya, Svea Giesecke, Nina Meißner, Matthias Seidel, Vincent Wyszynski, Julian Giesecke, Till Rech, Olaf Fritze, Jan Schildmann, Jochen Kreuder, Reinhard Hickel, Eckhart G. Hahn, Martin R. Fischer, Olaf Ahlers

**Affiliations:** 1 Medizinischer Fakultätentag der Bundesrepublik Deutschland e.V., Berlin, Germany; 2 Charité – Universitätsmedizin Berlin, Institut für Medizinische Informatik, LOOOP-Forschungsteam, Berlin, Germany; 3 Charité – Universitätsmedizin Berlin, Klinik für Anästhesiologie und Intensivmedizin CCM/CVK, LOOOP-Forschungsteam, Berlin, Germany; 4 Charité – Universitätsmedizin Berlin, Klinik für Neonatologie, Berlin, Germany; 5 Medizinische Hochschule Brandenburg Theodor Fontane, Fakultät für Gesundheitswissenschaften Brandenburg, Institut für Gesundheitswissenschaftliche Ausbildungsforschung, Neuruppin, Germany; 6 Universität Tübingen, Medizinische Fakultät, TIME – Tübingen Institute for Medical Education, Tübingen, Germany; 7 Universitätsmedizin Halle, Institut für Geschichte und Ethik der Medizin, Halle an der Saale, Germany; 8 Justus-Liebig-Universität Gießen, Institut für Hausärztliche Medizin, Gießen, Germany; 9 LMU München, Medizinische Fakultät, München, Germany; 10 Friedrich-Alexander-Universität, Medizinische Fakultät, Erlangen, Germany; 11 LMU Klinikum, LMU München, Institut für Didaktik und Ausbildungsforschung in der Medizin, München, Germany

**Keywords:** curriculum, competency-based medical education, frameworks, LOOOP

## Abstract

**Introduction:**

The “National Competence Based Catalogue of Learning Objectives for Undergraduate Medical Education” (NKLM) constitutes the content framework for medical studies in the Federal Republic of Germany. It defines the competencies that are deemed to be essential for all graduates to acquire by the conclusion of their studies, irrespective of the specialisation they subsequently pursue in residency. Another objective is to achieve what is termed “constructive alignment”, i.e. coordination between state examinations and faculty learning, teaching and assessment. This manuscript describes the development of the NKLM since 2009, with the overarching objective being the continual enhancement of the catalogue’s usability for the faculties.

**Project description:**

The creation and revision of the catalogue were conceptualised and coordinated in various constellations by the Association for Medical Education (GMA), the German Association of Medical Faculties (MFT), and the LOOOP research team at Charité and Brandenburg Medical School. The most recent findings from educational research have been continuously incorporated into the structure of the catalogue and the processing algorithms. In light of the aforementioned algorithms, the catalogue has been revised by a team of over 1,000 subject matter experts. The release of Version 2.1 is scheduled for the summer of 2026.

**Summary and outlook:**

The catalogue has undergone a series of refinements, improvements to its internal structure, and a substantial reduction in content scope across three interim versions: NKLM 1.0, NKLM 1.0 (neo), and NKLM 2.0. The NKLM serves as the foundation for the core curricula of the faculties. It is designed to be a living document that is continually adapted to new findings and topics. Development of the subsequent version, designated as 3.0, is already in preparation.

## Zusammenfassung

**Einleitung:**

Der Nationale Kompetenzbasierte Lernzielkatalog Medizin (NKLM) bildet das inhaltliche Rahmenwerk des Medizinstudiums in der Bundesrepublik Deutschland. Er definiert diejenigen Kompetenzen, die alle Absolvierenden am Ende des Studiums erworben haben sollen – unabhängig davon, in welcher Fachrichtung sie dann eine Weiterbildung beginnen. Angestrebt wird zudem das sogenannte „Constructive Alignment“, also insbesondere eine Abstimmung der Staatsexamina mit dem fakultären Lernen, Lehren und Prüfen. Das vorliegende Manuskript beschreibt den Entwicklungsprozess des NKLM seit 2009, dessen Ziel es war, die Nutzbarkeit des Katalogs für die Fakultäten immer weiter zu verbessern.

**Projektbeschreibung:**

Die Katalogerstellung und -überarbeitung wurde in verschiedenen Konstellationen durch die Gesellschaft für Medizinische Ausbildung (GMA), den Medizinischen Fakultätentag (MFT) sowie das LOOOP-Forschungsteam an Charité und Medizinischer Hochschule Brandenburg konzipiert und koordiniert. Es sind immer wieder aktuelle Ergebnisse der Ausbildungsforschung in die Struktur des Katalogs und in die Bearbeitungsalgorithmen eingeflossen, auf deren Grundlage insgesamt über 1000 (Fach-)Expert:innen die Kataloginhalte er- bzw. bearbeitet haben. Im Sommer 2026 soll die Version 2.1 veröffentlicht werden.

**Fazit und Ausblick:**

Über drei Zwischenversionen (NKLM 1.0, NKLM 1.0 (neo), NKLM 2.0) konnten der Katalog präzisiert, die interne Katalogstruktur verbessert und der inhaltliche Umfang schrittweise deutlich reduziert werden. Als Grundlage für die Kerncurricula der Fakultäten soll der NKLM ein lebendiges Werk sein, das immer wieder an neue Erkenntnisse und Themen angepasst wird. Die Erarbeitung der nächsten Version 3.0 ist bereits in Vorbereitung.

## 1. Introduction

The “National Competence Based Catalogue of Learning Objectives for Undergraduate Medical Education” (NKLM) is conceived as the foundational framework for competency-based medical training within the Federal Republic of Germany. The competency-based approach aligns with international concepts of Competency-Based Medical Education (CBME) [1], which have been established since the 2000s and are rooted in the principles of outcome-based training that emerged in the 1990s [2]. The overarching objective of CBME – and thus of the NKLM – is to ensure that all newly licensed doctors possess all the necessary and therefore comparable competencies at the commencement of their further training [1], [3], [4], [5].

**Figure 1:**
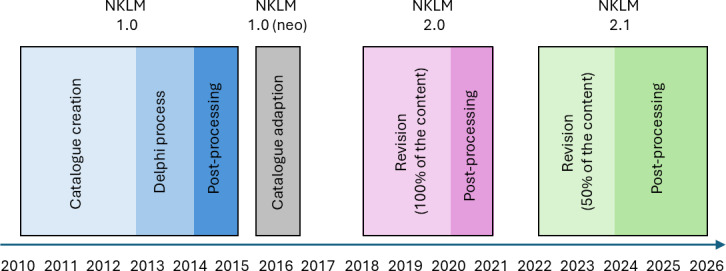
Overview of the stages of catalogue development across the various NKLM versions

The subsequent introduction offers a broad summary of the 15-year development process of the NKLM and its various interim versions (see also figure 1 (Fig. 1)). The project description subsequently delineates the development phases of the respective NKLM interim versions in detail. At the conclusion of each development stage, a synthesis of the salient findings from the pertinent work process is prepared. The results are integrated into a sub-section entitled “contents and catalogue presentation” in the project description to facilitate understanding. Consequently, a separate results section in the manuscript has been omitted. Due to the constraints imposed by the limitations of space, the text addresses only a few select aspects of the NKLM's structure and content. However, references and links are provided to enable access to further detailed information.

### 1.1. NKLM 1.0

The starting point for development of the NKLM was a request made in 2009 to the DACH Association for Medical Education (GMA) by the Higher Education Committee of the Standing Conference of the Ministers of Education and Cultural Affairs: The GMA, in consultation with the German Association of Medical Faculties (MFT), should develop a “professional qualification framework” for medical studies that would enable the development of a curriculum for a tiered study structure in the sense of bachelor's and master's degrees [6]. Arguing that a national core curriculum/catalogue of learning objectives for Germany was first necessary in order to fundamentally improve the quality of medical education and make it more comparable, the GMA’s pre-existing idea of creating a catalogue of learning objectives based on international frameworks was implemented [6], [7]. Under the joint coordination of GMA and MFT, the NKLM was developed based on the study sections defined in the Medical Licensure Act (ÄApprO) [8].

The NKLM version 1.0 was formally endorsed by a large majority in June 2015, together with the National Competence-based Learning Objectives Catalogue for Dentistry (NKLZ , which had been developed in parallel) at the annual meeting of the German Association of Medical Faculties (oMFT) in Kiel, and the faculties were encouraged to test the NKLM and NKLZ [7]. 

### 1.2. NKLM 1.0 (neo)

In 2016, the LOOOP research team [9] (hereinafter referred to as the “LOOOP team”) at Charité – Universitätsmedizin took the initiative to further develop NKLM 1.0 into a structurally adapted version, NKLM 1.0 (neo). The acronym “LOOOP” is derived from the “**L**earning **O**pportunities, **O**bjectives, and **O**utcomes **P**latform”, which was developed by the team. This platform functions as the central online instrument for work in the associated international LOOOP network for research in Health Sciences Education.

In 2017, Version NKLM 1.0 (neo) was made available online for medical faculties. The following link provides access: [https://nklm-10-neo.looop-network.org]. Key features of NKLM 1.0 (neo) were enhanced usability and navigability by optimizing the cross-references within the catalogue and facilitating the existing information’s accessibility (for details, see [10], [11]). The development of NKLM 2.0 was predicated on the foundation laid by NKLM 1.0 (neo).

### 1.3. NKLM 2.0

The “Master Plan for Medical Studies 2020” [12], adopted in 2017 by the Federal Ministry of Health and the Federal Ministry of Education and Research in conjunction with representatives of the conference of health and scientific ministers of the German states, outlined a reform of medical studies with a focus on competence orientation. The objective was to establish a comprehensive framework that integrates basic sciences with clinical content, a concept referred to as the “z-curriculum”. This initiative entailed the development of a modular, interdisciplinary curriculum, designed to facilitate a systematic and integrated learning experience across all semesters. Furthermore, the NKLM was to undergo additional development to serve as a nationally binding content basis for the core curricula of medical faculties. The specific design of the curricula, including teaching formats, teaching disciplines/ departments, and the sequence of learning content within study sections, remained the responsibility of the faculties. In addition to the core curriculum content of the NKLM, the faculties were expected to conceptualise approximately 25% of their teaching hours using own content. These teaching hours were to consist of elective (compulsory) courses and faculty-specific specialisations [13]. 

The subsequent evolution of NKLM version 2.0 was conceptualised and administered collectively by MFT and the LOOOP team as a component of a scientific collaboration initiated on June 1, 2018. This initiative enabled the utilisation of LOOOP’s preparatory efforts and conceptual frameworks by MFT [https://nklm.looop-network.org/objective/list/orderBy/@objectivePosition/modul/200563]. 

In accordance with the provisions outlined in the Master Plan for Medical Studies 2020 and the preliminary draft legislation for a new ÄApprO, which has been published by the German Federal Ministry of Health, it is recommended that the content of state examinations should be more closely aligned with the content of the medical degree programs. Consequently, the German institute for state Examinations in medicine, pharmacy, dentistry and psychotherapy (IMPP) opted to devise a “competence-oriented subject catalogue” (GK), drawing upon the NKLM 1.0 (neo) framework. In spring 2018, a few months before the development of NKLM 2.0 began, the IMPP also launched a scientific cooperation project with the LOOOP team. The first results of this project were then incorporated into the NKLM further development process.

The global COVID-19 pandemic had a significant impact on the availability of the experts involved in the process, necessitating the conversion of nearly all process steps to online formats. This shift in operations resulted in a six-month delay in the process. Consequently, NKLM 2.0 [https://nklm-20.looop-network.org/menu] was formally endorsed in March 2021, during an extraordinary meeting of the German Association of Medical Faculties (aoMFT), and was subsequently published in April 2021 [14].

### 1.4. NKLM 2.1 and NKLM 3.0

The NKLM 2.0 contained a number of formal inconsistencies and was considered by many faculties to be too comprehensive to fulfil the aforementioned function of a potentially binding basis for the core curricula at the faculties in a readily applicable manner. This notion has since been corroborated by a subsequent study [15]. Consequently, the scientific collaboration between MFT and the LOOOP team was prolonged, and a subsequent revision process was collaboratively conceptualised in the summer of 2021. This process was predicated on the anticipated entry into force of a new ÄApprO in autumn 2025, which was designed to provide the NKLM with legal force. To ensure adequate lead time for implementation at the faculties, the subsequent version of the NKLM was scheduled for adoption in 2023.

Given the impracticality of completing the entire NKLM in a timely manner with high quality in terms of content and structure by 2023, the further development of the NKLM was divided into two work packages. Work package 1 encompassed the NKLM content that would have been immediately necessary for the faculties to prepare for the new ÄApprO from 2023 onwards (for details, see 2.4.1); work package 2 correspondingly encompassed the remaining NKLM content. Given the absence of endorsement for any revised version of ÄApprO, the processing of work package 1 underwent a protracted, stepwise progression, extending over a period of approximately three years. This delay was implemented with the objective of allocating additional time to all involved parties. The subsequent interim version of NKLM (NKLM 2.1) is currently scheduled for release in the summer of 2026 and will afterwards undergo further development into version 3.0 (see figure 1 (Fig. 1)).

## 2. Project description

### 2.1. NKLM 1.0 – 2010 to 2015

#### 2.1.1. Introductory explanations

The development of the NKLM was a seminal moment for medical faculties, as it provided them with a novel opportunity to define the competencies and sub-competencies to be acquired during students’ studies. This initiative was consistent with a national recommendation, emphasizing a commitment to the recommendation of the content of medical education across the country. Furthermore, learning objectives were operationalized within the sub-competencies to create a comprehensive “library” for the faculties to test. Previously, the ÄApprO provided only broad regulations for the content of medical studies, which were supplemented by the IMPP subject catalogues. However, these subject catalogues were not competency-based and were exclusively focused on the written state examinations. Prior to its formal adoption, the pivotal function of the NKLM was formally recognized in the 2014 recommendations of the German Science and Humanities Council [16]. Consequently, it was expeditiously and favourably recognized as a pivotal opportunity to influence the national development of medical studies.

#### 2.1.2. Process design and implementation

The initiative was initiated with the establishment of 21 working groups (WGs) for the chapters of the NKLM, comprising over 200 experts, with the involvement of an additional pool of experts. These WGs reported to a GMA project group, which in turn presented its work results to a steering group (see fig. 1 in [6]). Following the initial development of the NKLM in this particular context, a two-stage Delphi consensus process was executed over the course of two years, adhering to the guidelines established by the association of scientific medical societies (AWMF). This process involved the participation of over 160 specialized societies within the AWMF [6], [7], [8].

The process, which was conducted primarily using office documents (e.g., Microsoft Word/Excel), received its impetus from an NKLM-Office situated within the purview of the chair of didactics and educational research in health care at the University of Witten/Herdecke. The NKLM-Office finalized the most recent iteration of NKLM 1.0, a process that entailed consultation with the MFT [8]. 

#### 2.1.3. Contents and catalogue presentation

The NKLM 1.0 [8] consisted of a total of 21 chapters: Four chapters with introductory information were followed by seven chapters describing medical roles based on the CanMEDS roles [17], followed by eight further chapters on medical competencies, sub-competencies and learning objectives, as well as one chapter on reasons for medical consultations and one on diseases. Various levels of competence were defined for learning objectives, which were then assigned to study sections in order to determine at what point in the course of study this level of competence should be achieved. In the case of diseases, the aspects were defined in which students were to acquire so-called “competency to act”. There were “cross-references” (CR) in many places between the different chapters – especially between diseases and learning objectives, but also between learning objectives themselves. These CR were supplemented by free text that linked the content [11]. The NKLM 1.0 was implemented and tested to varying degrees at several faculties [18], [19].

#### 2.1.4. Findings from the development of NKLM 1.0

On a positive note, it can be said that a complex process involving broad participation from the faculties and professional associations has been successfully completed with a high degree of consensus, resulting in the first version of the NKLM. However, the content of NKLM 1.0 was very extensive [20] and could not be significantly streamlined using the Delphi method, particularly because many representatives of professional associations focused on relevant specialist content. Notwithstanding the considerable dedication exhibited by all parties involved, a number of inconsistencies were identified in NKLM 1.0. Notably, the CRs were found to be incomplete, with discrepancies between the specified levels of competence and the verbs employed in the learning objective texts. Further details on this discrepancy can be found in [10] and [11]. Concurrently, the process was notably laborious and time-consuming due to the numerous committees and interactions involved. 

### 2.2. NKLM 1.0 (neo) – 2016

#### 2.2.1. Introductory explanations

A curriculum should be represented in an online “curriculum map” that transparently shows the content and how its components relate to each other [9], [21], [22]. This recommendation is consistent with the findings of the German Science and Humanities Council in 2014 [16], which underscores the importance of incorporating the principles outlined in this concept into the NKLM 1.0 (neo) framework.

The aim of such a “map” is to clearly show

1. *what *(here in the sense of operationalised learning objectives/“SMART” criteria [23], [24]),
2. *by whom* (here in the sense of subject recommendations),
3. *when or in what order* (here in the sense of study sections),
4. *in what context* (here in the sense of CR and free-text explanations),
5. *at what level of competence*

it should be learned [21]. Furthermore, the interests of various interest groups are not uniform when evaluating a curriculum. Therefore, the entire curriculum is subject to review only in extraordinary circumstances. More commonly, the focus is directed towards specific components of the curriculum and the relationships between them. Harden characterized this phenomenon as a series of “windows” through which one might have a look into a house, resulting in a continually shifting perspective [21]. In order to achieve a greater degree of alignment with these requirements, which were only partially fulfilled in NKLM 1.0, the catalogue underwent a structural enhancement to version NKLM 1.0 (neo) [https://nklm-20.looop-network.org/menu].

#### 2.2.2. Process design and implementation

The process was designed and carried out by the LOOOP team.

#### 2.2.3. Content and presentation

To enhance the thematic references within the NKLM, the CRs available in NKLM 1.0 were multiplied. The associated process was based on the analysis of information that was available in NKLM 1.0 as free text. This is described in detail elsewhere [10], [11]. In addition, the subject recommendations stored in NKLM 1.0 were prepared in such a way that they were explicitly visible for each learning objective.

To facilitate comprehension, the content was consolidated into a unified tabular presentation. The original CRs were rendered in plain text, and all supplemented CRs were appended directly subsequent to the free texts. By clicking on the CR, users can navigate through the catalogue and visualize the references within the NKLM in detail [https://nklm-10-neo.looop-network.org/intralinks]. Additionally, a wide range of filter, search, and export functions were at the user's disposal, thereby empowering them to selectively observe particular components through the designated “windows”.

#### 2.2.4. Findings from the development of the NKLM 1.0 (neo)

Points 2 and 4 from section 2.2.1 were successfully integrated through the enhancement of the representation of the relationships between chapters, thereby facilitating the recognition and utilization of the assigned subjects. As the content orientation of NKLM 1.0 was not to be changed, this was not possible for points 1, 3 and 5 from section 2.2.1, which were therefore only addressed in the further development of NKLM 2.0.

### 2.3. NKLM 2.0 – 2018 to 2021

#### 2.3.1. Introductory explanations

A primary objective of the revision to NKLM 2.0 was to reduce the content: The term “core curriculum-relevant” was defined as follows: It refers to the subject matter that doctors will regularly encounter at the beginning of their future work, regardless of the further training they subsequently choose to pursue. 

The remaining concepts mentioned in section 2.2.1, which were not yet implementable in NKLM 1.0 (neo), were fully incorporated into both the process and the NKLM itself by the LOOOP team for NKLM 2.0. In addition to points 1 to 5 of the map delineated in 2.2.1, these concepts encompassed versioning of NKLM content and transparent documentation of changes (predecessor-successor relationships, change documentation). In addition, the preexisting LOOOP rights and process control system was integrated into the process, thereby facilitating the concurrent synchronous and asynchronous editing of content on the online platform. This approach fosters optimal transparency, thereby enabling real-time intervention at every stage of the editing process. It is noteworthy that all participants possess the capacity to observe and modify the content in real time, thereby ensuring a dynamic and collaborative environment. Furthermore, the congruence between the verbs employed in the learning objectives and the designated levels of competence was ensured by implementing LOOOP’s taxonomy [9], [15], [25], [26] into the process. This taxonomy has been developed since 2004 by the LOOOP team. It represents aspects of modified Bloom's taxonomy [27] and the Miller pyramid [28] and now also serves as the foundation for online learning objective processing in the NKLM. For a more comprehensive overview, please refer to tab. 1 in [26]. In order to enhance clarity, the NKLM was presented in a reduced number of columns.

#### 2.3.2. Process design and implementation

In accordance with the “Master Plan for Medical Studies 2020”, an NKLM commission – headed by the MFT-president – was established with representatives from the federal and state governments (ministries of science and health), IMPP, MFT and GMA, as well as other guests (including the AWMF and the federal representation of medical students in Germany). A new NKLM-Office was set up at the MFT, which was established and headed by the coordinator of the LOOOP team. Both the design and implementation of the NKLM development process were jointly ensured by the MFT and the LOOOP team.

**Figure 2:**
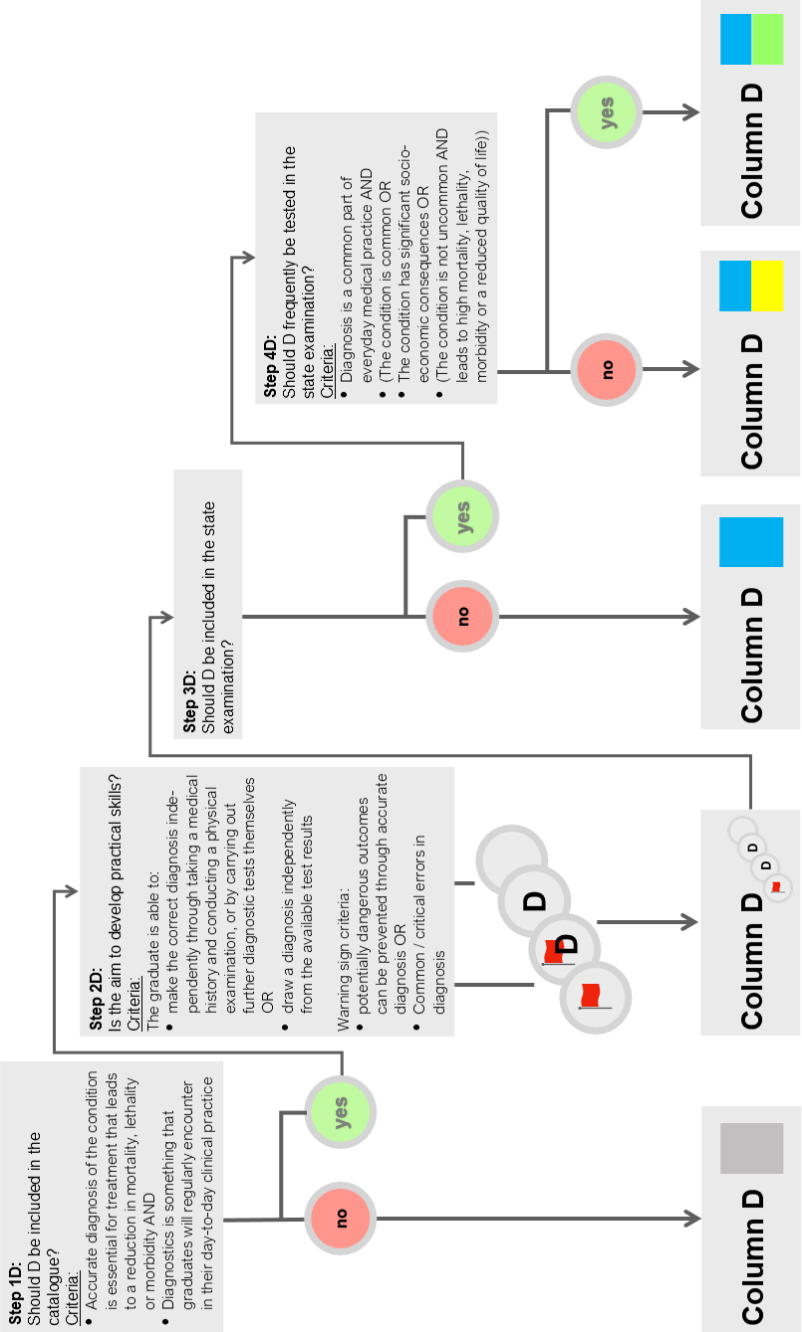
Example algorithm for defining the “diagnostics” column for the joint development process of NKLM 2.0 and the object catalogue (GK) D: Diagnostics, blue: marker for NKLM, green/yellow: marker for GK. Red flags were raised when incorrect diagnoses could potentially have serious consequences for those affected

This process was intentionally designed to be participatory: approximately 800 experts, mainly from faculties and professional associations, worked under the close supervision of the NKLM-Office in 25 interdisciplinary (and in some cases interprofessional) WGs on the content of each NKLM chapter, based on the content of NKLM 1.0 (neo). The NKLM commission coordinated the process. Despite the increased number of experts, the process was thus significantly streamlined compared to NKLM 1.0. The process was based on algorithms for the combined processing of NKLM and GK [https://nklm.looop-network.org/objective/list/orderBy/@objectivePosition/modul/200557]. An example of a combined NKLM-GK decision algorithm is shown in figure 2 (Fig. 2). The algorithm always started with the question of the relevance of the respective NKLM aspect (in this case, diagnostics). Subsequently, the depth of competence for the NKLM was first determined, and then the depth of competence for the GK was determined on this basis. Based on these algorithms, the NKLM-Office created work instructions for the experts and further refined them during the process, which is described online [https://nklm.looop-network.org/objective/list/orderBy/@objectivePosition/modul/200563]. The content for NKLM 2.0 was edited exclusively online in the LOOOP online platform in order to avoid additional editing time due to the export and re-import of data as well as transmission errors.

#### 2.3.3. Content and presentation

The NKLM 2.0 consisted of eight main chapters (I to VIII) with various subchapters. In addition, three lists of medicinal substances, pathogens and excerpts from medical law were compiled. For each piece of content, in addition to the depth of competence already available in NKLM 1.0, the SMART criteria were used to clearly define what was to be learned. This definition was formulated in conjunction with the learning objective text (employing standardised verbs) through supplementary CR [11], which included “clarifications” and “additional explanations”. All this information has now been condensed into a few columns. In a separate LOOOP-AWMF collaborative endeavour, AWMF specialists assigned all NKLM content (with the exception of chapter V “reasons for consultation”) to disciplines in the sense of recommendations. These recommendations were viewable in a separate column. Due to the many references within the catalogue and the comprehensive filter and search options, NKLM 2.0 was only available online. Information on the structure of NKLM 2.0 can be found here: [https://nklm.looop-network.org/objective/list/orderBy/@objectivePosition/modul/200566].

#### 2.3.4. Findings from the development of NKLM 2.0

A substantial advancement was achieved in the clarification and reduction of the NKLM content, a subject that is thoroughly delineated in [11]. Nevertheless, the considerable involvement of experts and the open process design resulted in heterogeneous participation in the working group meetings. This necessitated substantial support from the NKLM-Office to ensure that all parties involved were up to date. A considerable proportion of these meetings were self-organized by the experts, who possessed the capability to edit the platform independently. Notwithstanding, this methodology also resulted in heterogeneous editing of the NKLM content on the online platform. Consequently, at the conclusion of the process, the NKLM-Office was obliged to undertake time-consuming post-processing in order to achieve approximately homogeneous editing of the various chapters and internal consistency of the catalogue. In this phase of post-processing, the content underwent adaptation in terms of content and editing by the NKLM-Office staff, who were generally medical professionals. The resultant data were subsequently disseminated in smaller packages to the working groups for final review and approval. This approach by the NKLM-Office was considered by all participants to be effective and efficient. A further limitation emerged from the chapter-by-chapter operational framework of the working groups, which resulted in inadequate coordination among them. Consequently, the network of CRs lacked comprehensive coordination between the working groups, resulting in unclear thematic links between the content. However, due to the demands of other essential tasks, the NKLM-Office was unable to optimize these references during the subsequent phase as well [11].

The aforementioned experiences yielded several insights that informed the subsequent revision process.

- It is recommended that the number of working groups and the number of volunteer experts involved be reduced. The implementation of fixed group compositions, accompanied by the establishment of a quorum for decision-making processes, is recommended.
- The content should no longer be worked on by the groups within a single chapter. Instead, a collective approach is recommended for the management of topics, with due consideration for the CR.
- It is imperative that all working meetings of experts are supervised by the NKLM-Office, functioning as an “interface” to facilitate the requisite continuous coordination between the various working groups and chapters.

### 2.4. NKLM 2.1 (2022 to 2026)

#### 2.4.1. Introductory explanations

The subsequent NKLM 2.1 development process encompassed the content of the introductory work package 1 (approximately 50% of the NKLM 2.0 content). This was preceded by an online evaluation process conducted by the medical faculties, which was jointly designed and implemented by MFT and the LOOOP team. The results of this process were taken into account in the development of NKLM 2.1. The subject of work package 1 was

- all content that, according to the draft of the new ÄApprO, should have a clinical relevance in the first part of the z-curriculum,
- all content for which a reference to the topics of “patient safety” or “digital skills” had been established in NKLM 2.0,
- all overarching competencies in chapter VIII (e.g. interprofessional or scientific competencies).

#### 2.4.2. Process design and implementation

**Figure 3:**
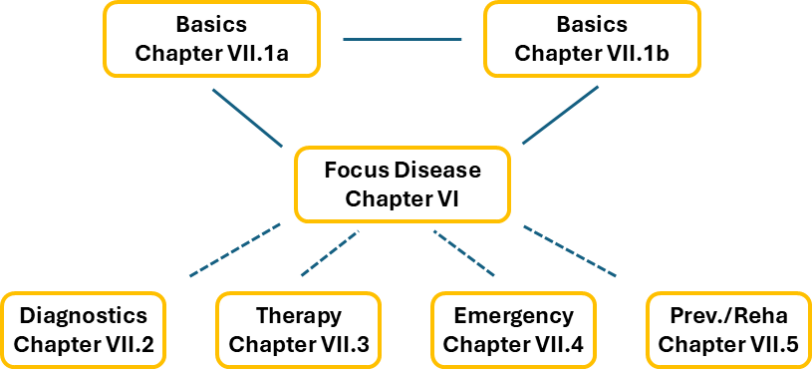
Example of an algorithm for defining the “focus network” for NKLM 2.1 Cross-references marked by solid lines must always be present; cross-references marked by dotted lines may be present – at least one cross reference marked by dotted line is mandatory for each focus disease.

The NKLM 2.1 process was again developed in collaboration by the MFT and the LOOOP team, with the LOOOP team providing ongoing support for the content-related tasks. The MFT was responsible for executing the process, while the LOOOP team provided ongoing support through the platform. A total of 100 experts were recruited to participate in 11 focus groups (FGs), which were established to address work package 1 [29]. Within each FG, a quorum of five individuals was established. New work instructions for the experts were created to support the FG’s work. Figure 3 (Fig. 3) shows an example of the interaction in the first section of the z-curriculum. The FG received consistent support from full-time or trained student staff from the MFT-Office during all meetings. Their involvement shifted from comprehensive chapter work to addressing content-related issues across various chapters. This addressed the points mentioned in section 2.3.4 under 1. to 3. 

A comprehensive revision of work package 1 was undertaken, encompassing both the content and the structure. Work package 2, will only be revised in terms of content for NKLM 3.0, but was also revised in terms of structure in order to ensure formal consistency within NKLM 2.1. Further information on the process can be found on the MFT website [30].

#### 2.4.3. Content and presentation

A comprehensive revision of the content of NKLM 2.1 has been undertaken. This revision has involved a reduction in the volume of content, a rearrangement of the content of many chapters, and the active removal of redundancies from the catalogue. The scope of the CR network has been reduced and the network has at the same time been completed to define the thematic connections [11]. The presentation of NKLM 2.1 has undergone no structural changes in comparison with NKLM 2.0. However, certain visual enhancements have been implemented, resulting in a substantial increase in readability and clarity. This enhancement was achieved by decreasing the number of CRs, clarifications, and additional explanations. The NKLM 2.1 will be disseminated in two versions: One version is based on the study sections from the latest draft bill for a new ÄApprO (1st study section: semesters 1 to 6, 2nd study section: semesters 7 to 10), and the other version has been adapted to the currently valid ÄApprO (1st study section: semesters 1 to 4, 2nd study section: semesters 5 to 10). The allocation of content to the respective study sections will differ between the two versions; however, the content itself will remain consistent.

Furthermore, plans are underway to develop a compact version of the NKLM for individuals who are interested in the system but do not require a comprehensive exploration of its intricacies. This version will contain a more concise representation of the NKLM, facilitating an accessible introduction to its principles.

#### 2.4.4. Findings from the development of the NKLM 2.1

This subsequent version of the NKLM was also significantly enhanced in terms of precision and further reduction of content [11]. The FG’s efforts were facilitated by the reduced number of experts. Nevertheless, significant challenges were encountered in attempting to reach a quorum. 

The outcome of the FG’s efforts exhibited greater heterogeneity compared to that observed with NKLM 2.0. Consequently, the duration of the subsequent phase, referred to as the “post-processing phase”, had to exceed that of the initial "processing phase”. Furthermore, the subsequent phase necessitated a substantial allocation of resources. Consequently, a number of the planned content revisions for work package 1 were deferred to the ensuing NKLM 3.0 processing sequence. The follow-up itself was carried out according to a similar principle as for NKLM 2.0: the MFT-Office developed proposed changes, which were agreed upon by the FG (spokespersons). As was the case with NKLM 2.0, this approach was regarded as highly effective by many participants.

The following insights for the subsequent revision process were gained from the experiences mentioned above:

1. Due to the limited time available, the mode of expert work should be reconsidered and optimised in terms of time efficiency.
2. The role of the MFT-Office should be strengthened and the process made more effective in the style of the post-processing phase of NKLM 2.1. In this model, processing proposals are prepared by the MFT-Office and then commented on, discussed, and decided upon individually in a circulation procedure. The final decision is made jointly in the focus group meetings.

## 3. Outlook for the NKLM 3.0 process and conclusion

### 3.1. Outlook for the NKLM 3.0 process

The development of NKLM 3.0 will be based primarily on the content revision of work package 2 – supplemented by those elements of work package 1 that could not be addressed in NKLM 2.1 as originally planned. The experiences gained from the development of NKLM 2.0 and NKLM 2.1 will be critically reviewed in order to adapt the work process for NKLM 3.0.

Two aspects are particularly important here: firstly, the inconsistency in the processing results for both NKLM 2.0 and NKLM 2.1; and secondly, the longer processing time for NKLM 2.1 compared to NKLM 2.0. The main causes of these problems lay in the organisation of the work process: For instance, when compiling NKLM 2.0/2.1, the experts often still worked directly on the LOOOP online platform using the “native” text from the respective previous catalogue version. It may have been particularly challenging to take into account not only the content but also the formal criteria for the new version of the NKLM that was to be developed. In future, the MFT-Office will be able to present clear proposals for content revisions that have already been adapted to the formal aspects, which will then be discussed and agreed upon by the experts. This means that the experts will no longer have to navigate the complex online interface themselves, thereby eliminating a further source of errors and inconsistencies.

Another challenge is the division of the work packages. During the development of NKLM 2.0, the working groups worked largely autonomously on their respective chapters, coordinating with other working groups only on an ad hoc basis. For the development of NKLM 2.1 – as described here – two major work packages were initially formed, of which only the first has been revised so far; work on the second is still pending for NKLM 3.0. For the actual work within the working groups for NKLM 2.1, the content was further divided. This division was necessary to make the work process more manageable and to make appropriate use of the expertise of all those involved. However, this approach also led to increased coordination and consultation efforts, and an overall view of the chapters was difficult due to the topic-specific division. In future, the MFT-Office will play an even stronger coordinating role in this regard.

The workflow for NKLM 2.1 had to be adjusted several times. In addition, the steps involved in the FG meetings were also changed on several occasions. These changes were intended to optimise the ongoing process (e.g. to improve coordination of work between the FG meetings or to make better use of time during the meetings).

Overall, it is clear that, on the one hand, broad participation by a wide range of experts and stakeholders is important in order to ensure the greatest possible acceptance of the final catalogue. On the other hand, the process must be well prepared, coordinated and communicated in terms of both content and form by a central body – i.e. the MFT-Office – so that the work processes remain manageable.

The evaluation of NKLM 2.1 will also be essential for the further process leading to NKLM 3.0, particularly with regard to its usability by colleagues in the faculties. To this end, a multicentre study within the German LOOOP network is starting in summer 2026.

Even after the publication of version 3.0, the NKLM is to be continuously developed in close coordination and alignment with the content of the state examinations. In doing so, the NKLM must remain a living document that is regularly adapted to new medical and scientific findings and important socio-political issues. For example, adjustments have already been made to the catalogue in the wake of the coronavirus pandemic. 

With a view to the long-term development of the NKLM, it is expected that version 3.0, which is significantly reduced in complexity and scope, will also streamline future processes considerably and enable shorter revision cycles.

### 3.2. Conclusion

The successive iterations of the NKLM have contributed to the reduction and refinement of the catalogue, a process that has occurred over the course of several years. Additionally, the internal catalogue structure and its usability have undergone substantial enhancement. In the process, the complexity of the NKLM has been significantly reduced compared to NKLM 2.0.

It should be particularly emphasised that, thanks to broad participation, the NKLM combines three essential points: improvement of competence-oriented teaching at medical faculties, improvement of the alignment of faculty teaching with the requirements of state examinations, and compatibility with specialist medical training. 

The findings from the analysis described in section 3.1 will be of significant benefit to the future process design for the development of version 3.0.

## Notes

### Funding 

At the request of the GMA, the Robert Bosch Foundation provided generous project funding for the financing of the NKLM-Office for the period from May 2010 till March 2012. After that, further revisions up to NKLM 2.1 were financed by MFT and the LOOOP team from their own resources.

### Authors’ ORCIDs

- Jaqueline Jennebach: [0009-0006-1572-8725]
- Julian Özkaya: [0009-0006-4691-829X]
- Svea Giesecke: [0009-0006-1925-0028]
- Nina Meißner: [0000-0002-3266-9206]
- Matthias Seidel: [0009-0007-5450-262X]
- Vincent Wyszynski: [0009-0007-2157-4352]
- Julian Giesecke: [0009-0003-6242-9443]
- Till Rech: [0000-0002-7451-9038]
- Olaf Fritze: [0000-0002-3825-3703]
- Jan Schildmann: [0000-0002-5755-7630]
- Joachim Kreuder: [0000-0001-6343-754X]
- Reinhard Hickel: [0000-0001-9185-6602]
- Eckhart G Hahn: [0000-0003-3261-0105]
- Martin R Fischer: [0000-0002-5299-5025]
- Olaf Ahlers: [0000-0003-1528-7182]

## Acknowledgements

The authors would like to thank

- the *many highly committed experts*, represented here by the (deputy) WG and FG spokespersons of the NKLM 2.0 and 2.1 process: Martin Aringer, Bettina Baeßler, Erika Baum, Stefan Beckers, Anja Bittner, Katrin Borucki, Beate Brand-Saberi, Georg Breuer, Stefan Bushuven, Gerhard Danzer, Ulrich Decking, Nadine Dreimüller, Martin Dugas, Matthias Eyrich, Götz Fabry, Nicolas Feltgen, Helmut Fickenscher, Stefan Frantz, Susanne Fröhlich, Annette Fröhmel, Volker Harth, Ruth Hecker, Gunther Hempel, Anne Hermann-Werner, Caroline Herr, Thorsten Hornung, Jana Jünger, Ortrud Karg, Claudia Kiessling, Uwe Kornak, Anika Krochmann, Heike Kölbel, Michael Kühl, Frank Lammert, Hanns-Martin Lorenz, Jens Lutz, André Mihaljevic, Eckard Nagel, Marcus Neudert, Markus Parzeller, Dorothea Penders, Alexandra Preisser, Bernd Romeike, Nicolas Schlegel, Andrea Schmedding, Rudolf Schubert, Sasa Sopka, Sandra Steffens, Bernhard Steinweg, Christoph Stosch, Ute Teichert, Peter Tinnemann, Rolf-Detlef Treede, Hartmut Vatter, Marcel Verhoff, Thomas Vogl, Wilfried Wagner, Christiane Waller, Jens Waschke, Tobias Weberschock, Julia Welzel, Boris Wittekindt;
- the *representatives of the AWMF, the bvmd, the GMA and the IMPP * who have supported the process over the years;
- Martina Kadmon, Matthias Frosch, Frank Wissing, as well as the other members of the MFT *Presidium and MFT Teaching Committee* and the former (student) *employees of the NKLM-Office*: Nasrin El-Bandar, Milena Höcht, Lea Poewe, Nathiesan Selvalingam, Philipp Tosberg;
- the other members of the *NKLM-accompanying committees* of the MFT: Annette Becker, Kirsten Gehlhar, Andreas Guse, Lutz Hein, Benita Sahyoun, Thorsten Schäfer, Blanche Schwappach-Pignataro;
- Our colleagues at the *GMA and NKLM offices for NKLM 1.0*: Beate Hespelein, Karin Mohn, Daniel Bauer;
- the current and former *members of the LOOOP team* at Charité and MHB, as well as the *institute for research in health sciences education* at the Faculty of Health Sciences Brandenburg: Tim Achterkamp, Franziska Louisa Arnold, Felix Balzer, Josephine Becker, Andreas Bietenbeck, Aviva Sugar Chmiel, Constanze Czimmeck, Martin Dittmar, Simon Drees, Lea Fieth, Martin Gavrysh, Inga Hege, Eike Christian Kühn, Michael Kuth, Lars Lehmann, Huy Le Duc, Robert Müller, Viola Niehoff, Marc Penecke, Iloise Ras, Tamara Pace Ross, Luis Salazar, Anna Schilli, Rebecca Schleiernick, Mary Showstark, Scott Smalley, Firman Sugiharto, Ina Treadwell, David Paul Weber, as well as the many colleagues from the member faculties of the international LOOOP training research network.

Without the ideas and support of these individuals and groups, the developments described here would not have been possible.

## Competing interests

The authors declare that they have no competing interests.
